# Distal anastomotic new-entry tear after type A aortic dissection repair: Incidence patterns and long-term outcomes by extent of aortic replacement

**DOI:** 10.1016/j.xjon.2025.09.018

**Published:** 2025-09-22

**Authors:** Go Yamashita, Shingo Hirao, Koh Yaegashi, Takumi Takauchi, Atsushi Sugaya, Jiro Sakai, Tatsuhiko Komiya

**Affiliations:** Department of Cardiovascular Surgery, Kurashiki Central Hospital, Kurashiki, Japan

**Keywords:** acute type A aortic dissection, distal anastomotic new-entry tear, long-term outcome, supra-aortic branch dissection

## Abstract

**Objective:**

The study objective was to investigate the incidence patterns, anatomic distribution, and long-term outcomes of distal anastomotic new-entry tear in acute type A aortic dissection according to the extent of aortic replacement.

**Methods:**

This retrospective study analyzed 409 patients with acute type A aortic dissection who underwent surgical repair between 2003 and 2023. Patients were categorized by the extent of aortic replacement: hemiarch replacement (n = 173), partial arch replacement (n = 126), and total arch replacement (n = 110). Distal anastomotic new-entry tear was identified using postoperative contrast-enhanced computed tomography. Long-term outcomes were distal anastomotic new-entry tear status (distal anastomotic new-entry tear vs nondistal anastomotic new-entry tear) and the extent of aortic replacement (hemiarch replacement, partial arch replacement, and total arch replacement).

**Results:**

Distal anastomotic new-entry tears occurred in 27.4% of the patients, predominantly in the greater curve of aortic arch across all replacement types. Supra-aortic branch dissection was an independent risk factor for distal anastomotic new-entry tear occurrence (odds ratio, 2.80, *P < .*001). Patients with distal anastomotic new-entry tear were younger and predominantly male. Long-term survival was similar between the distal anastomotic new-entry tear and nondistal anastomotic new-entry tear groups; distal anastomotic new-entry tear significantly increased the cumulative incidence of distal aortic reoperation, particularly in patients with hemiarch replacement and partial arch replacement, but not in those with total arch replacement. Multivariate analysis identified distal anastomotic new-entry tear (hazard ratio, 3.95, *P < .*001) and Marfan syndrome (hazard ratio, 7.46, *P =* .005) as independent predictors of distal reoperation.

**Conclusions:**

Distal anastomotic new-entry tear commonly develops at the greater curve of the aortic arch after acute type A aortic dissection repair and substantially increases the risk of distal reoperation after hemiarch replacement and partial arch replacement but not total arch replacement, suggesting that total arch replacement may protect against distal anastomotic new-entry tear-related reoperations, particularly in patients with supra-aortic branch dissection. Careful surgical planning and long-term surveillance are crucial for optimizing outcomes.

## Graphical Abstract

**Figure:**
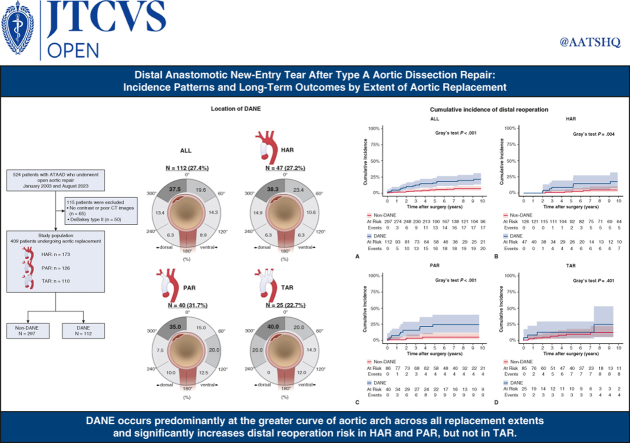

**Figure:**
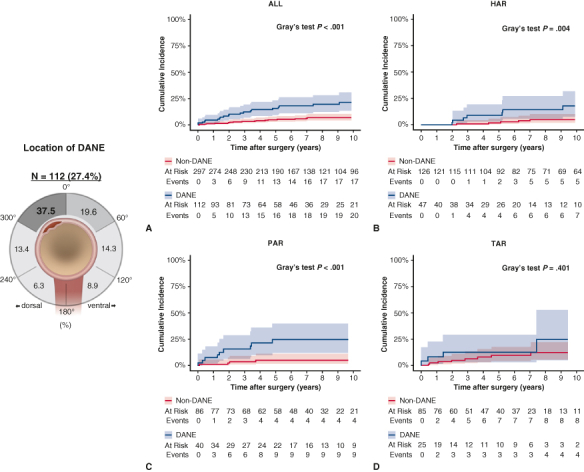
Predominant DANE occurrence at 301° to 360° and varying reoperation risks by repair extent.

**Central Message**

DANE preferentially develops at the greater curve of aortic arch (301°-360°) after ATAAD repair. It raises reoperation risk in HAR and PAR but not TAR; thus, individualized surgical planning is needed.

**Perspective**

DANE tends to occur at the greater curve of aortic arch across all replacement types, but increases reoperation risk only in HAR and PAR, not TAR. Supra-aortic branch dissection is an anatomic predictor of DANE. By demonstrating differential reoperation risks based on surgical extent, this work provides objective criteria for individualized surgical planning in ATAAD to avoid unnecessary reoperation.

Acute type A aortic dissection (ATAAD) is a life-threatening cardiovascular emergency requiring immediate surgical intervention. Despite the high mortality risk, in-hospital mortality rates have steadily improved over the past 2 decades.^1^^,^^2^ The fundamental surgical principle involves resection of the primary entry tear, with the extent of aortic arch replacement tailored to the entry tear location.^3^

Distal anastomotic new-entry tears (DANEs) pose a significant challenge regardless of the extent of replacement. They perpetuate false lumen perfusion, potentially compromising long-term aortic remodeling and patient outcomes.^4^ However, current evidence regarding DANE's long-term implications remains limited and predominantly focuses on hemiarch replacement (HAR),^5^ whereas the specific anatomic distribution of DANE occurrence remains inadequately characterized. This study elucidates the incidence patterns, anatomic predilection, and predictive factors for DANE formation stratified by the extent of aortic replacement and determines its long-term impact on distal aortic reoperation across different surgical approaches.

## Materials and Methods

The study adhered to the Declaration of Helsinki and its subsequent amendments. This retrospective observational study was conducted at a single center and was approved by the Institutional Review Board of Kurashiki Central Hospital (approval 4530; November 19, 2024). The requirement for informed consent was waived because of the retrospective nature of the study.

### Study Design and Patient Selection

A total of 524 patients with ATAAD who underwent open aortic repair at the Kurashiki Central Hospital between January 2003 and August 2023 were enrolled. We excluded patients who did not undergo contrast-enhanced computed tomography (CT) imaging within 6 months postoperatively, those with inadequate image quality for proper evaluation, and those who underwent DeBakey type II dissection. After excluding 115 patients (65 with inadequate CT image quality and 50 with DeBakey type II dissection), 409 patients were included.

**Figure 1:**
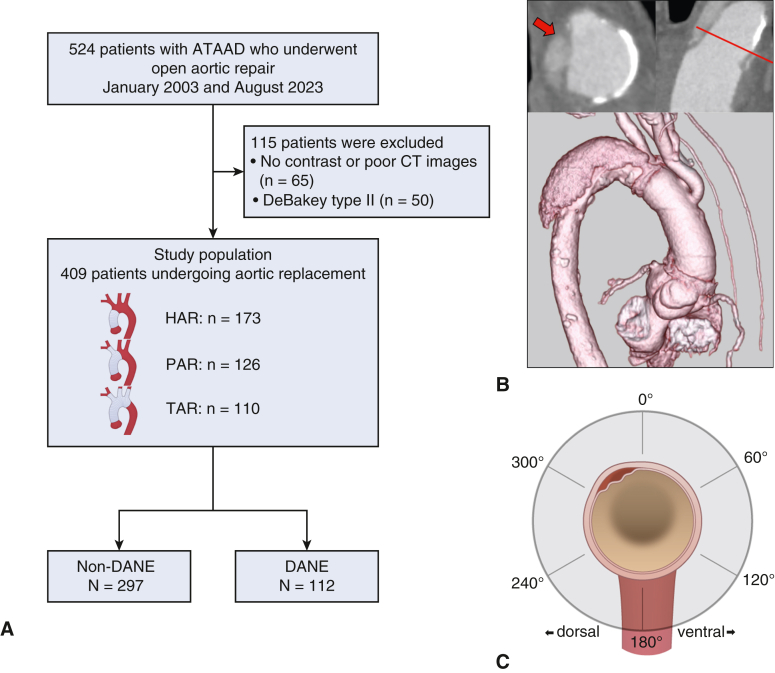
A, Patient selection flowchart illustrating the study population stratification by the extent of aortic replacement (HAR, PAR, TAR) and DANE status. B, Top: Contrast-enhanced CT images demonstrating DANE (*red arrow*) with blood-flow communication from the true lumen to the false lumen. Bottom: Three-dimensional CT reconstruction showing the relationship between DANE and the surrounding aortic anatomy. C, Schematic representation of the angular measurement system used to define the DANE location, with the 0° reference point varying according to the extent of aortic replacement.

To comprehensively evaluate the impact of the extent of aortic replacement on the occurrence and outcomes of DANE, we stratified the study population into 3 groups based on the surgical approach: HAR (n = 173), partial arch replacement (PAR, n = 126), and total arch replacement (TAR, n = 110). PAR included zone 1 and 2 reconstructions according to the Ishimaru classification. Furthermore, the entire cohort was classified into non-DANE (n = 297) and DANE (n = 112) groups for a comparative analysis (Figure 1, *A*). Data including patient characteristics, operative details, and outcomes were collected from electronic medical records, outpatient clinic visits, and telephone follow-ups for nonattendees. The follow-up rate was 94.4%.

### Definition and Location of Distal Anastomotic New-Entry Tear

DANE was defined as the communication between the true and false lumens at the distal anastomosis site identified using contrast-enhanced CT with multiplanar reconstruction. In this evaluation, the axis was aligned with the cross-section of the anastomosis site and carefully shifted along the parallel axis to confirm the continuity of blood flow into the false lumen (Figure 1, *B*). Blood flow reentering the false lumen from the supra-aortic branches more than 2 cm distant to the anastomosis was not classified as DANE in this study, because tears at incorporated branch ostia were considered suture-related injury rather than preexisting dissection. CT images were independently reviewed by board-certified cardiovascular surgeons (G.Y. or A.S.) and analyzed in the early arterial phase using a specialized workstation (syngo.via, Siemens). To characterize the precise location of DANE, we established an angular measurement system that defined the positions in a clockwise direction. The 0° reference point varied according to the extent of the aortic replacement. For HAR, the innominate artery was used as the reference point: PAR for left carotid or subclavian artery and TAR for replaced left subclavian artery (Figure 1, *C*). All measurements were performed in a blinded manner.

### Surgical Techniques

The detailed surgical techniques have been reported.^6^^,^^7^ The turn-up anastomosis technique, as previously described, involves placing 5 to 8 pairs of U-stay 4-0 polypropylene sutures at a depth of 1 cm with external felt reinforcement. After tying these sutures to evert the graft edge, continuous 4-0 polypropylene suturing is performed—beginning at the lesser curvature for HAR or at the 12-1o'clock position (surgeon's view) for PAR/TAR. This technique maximizes the anastomotic surface area and ensures excellent hemostasis. Distal anastomosis was performed using the turn-up anastomosis technique in 99.8% (408/409) of the cases. The use of biological glue at the distal anastomosis site has evolved over time; it was routinely used during the 2000s, gradually reduced in the late 2010s, and completely discontinued as of 2020. The reconstruction sequence also underwent temporal modifications. Initially, the ascending aorta was routinely clamped to prioritize proximal anastomosis, whereas in recent years, we have transitioned to performing distal anastomosis first under circulatory arrest without ascending aortic clamping. For cerebral protection, we used antegrade selective cerebral perfusion via individual cannulation of the supra-aortic branches.

### Statistical Analysis

Continuous variables are presented as medians (interquartile range [IQR]) and were compared using the Mann–Whitney *U* test. The normality of the data was assessed using the Shapiro–Wilk test. Categorical variables are presented as counts and percentages and were compared using the Fisher exact test. Long-term survival was analyzed using the Kaplan–Meier method with log-rank testing, and the cumulative incidence of distal aortic reoperation was evaluated using competing risk analysis with a Fine-Gray model, considering death as a competing event. The odds ratios (ORs) of risk factors for DANE—including age, sex, body surface area, Marfan syndrome, use of distal glue, HAR, PAR, ascending aortic clamping, and supra-aortic branch dissection—were calculated using a multivariable logistic regression model. Hazard ratios (HRs) of risk factors for distal aortic reoperation based on age, sex, Marfan syndrome, use of distal glue, TAR, elephant trunk (ET), supra-aortic branch dissection, and DANE were calculated using a Cox proportional hazards model. No collinearity was found between the risk factors used in these models as assessed by the variance inflation factor less than 5 for all variables. All statistical analyses were performed using R statistical software version 4.4.1 (The R Foundation for Statistical Computing).

## Results

### Patient Characteristics and Intraoperative Variables

Patients in the DANE group were significantly younger (median 65 vs 70 years, *P =* .012) than those in the non-DANE group and predominantly male (59.8% vs 45.8%, *P =* .015). Body surface area was higher in the DANE group (1.70 vs 1.62 m^2^, *P =* .057). Although the prevalence of comorbidities remained comparable between the groups, the DANE group showed significantly higher rates of severe preoperative aortic valve regurgitation (11.6% vs 4.0%, *P =* .009), cerebral malperfusion (20.5% vs 7.4%, *P < .*001), lower-extremity malperfusion (18.8% vs 9.1%, *P =* .009), and supra-aortic branch dissection (81.2% vs 58.2%, *P < .*001) (Table 1). The turn-up anastomosis technique was used in nearly all patients (99.6% in the non-DANE group vs 100% in the DANE group, *P* > .99). Biological glue use at the distal anastomosis was similar between groups (48.5% vs 48.2%, *P* > .99). The DANE group had longer operative times (371 vs 339 minutes, *P =* .03) and a higher volume of intraoperative bleeding (2090 vs 1426 mL, *P < .*001). The distribution of the extent of aortic replacement was similar between the groups (Table 2).

**Table 1:** Patient characteristics

Variable	Non-DANE group	DANE group	*P* value
	N = 297	N = 112	
Age, y	70 [59-79]	65 [57-74]	.012
Sex (male)	136 (45.8)	67 (59.8)	.015
Body surface area, m^2^	1.62 [1.48-1.78]	1.70 [1.48-1.87]	.057
Body mass index, kg/m^2^	23.2 [20.8-25.5]	23.4 [21.0-26.1]	.46
Hypertension	220 (74.1)	77 (68.8)	.32
Hyperlipidemia	57 (19.2)	21 (18.8)	>.99
Smoking	107 (36.0)	40 (35.7)	>.99
Diabetes	16 (5.4)	5 (4.5)	.807
CKD (eGFR <45, mL/min/1.73 m^2^)	71 (23.9)	26 (23.2)	>.99
Previous stroke	27 (9.1)	14 (12.5)	.356
Marfan syndrome	2 (0.7)	2 (1.8)	.302
Cardiac tamponade	63 (21.2)	16 (14.3)	.124
Cardiogenic shock	46 (15.5)	14 (12.5)	.532
Cardiopulmonary resuscitation	7 (2.4)	2 (1.8)	>.99
Preoperative intubation	11 (3.7)	7 (6.2)	.283
Preoperative aortic valve regurgitation			
None or trace	181 (60.9)	58 (51.8)	.115
Mild	85 (28.6)	30 (26.8)	.805
Moderate	19 (6.4)	11 (9.8)	.287
Severe	12 (4.0)	13 (11.6)	.009
Malperfusion			
Coronary	18 (6.1)	7 (6.2)	>.99
Cerebral	22 (7.4)	23 (20.5)	<.001
Visceral	24 (8.1)	9 (8.0)	>.99
Lower extremity	27 (9.1)	21 (18.8)	.009
Supra-aortic branch dissection	173 (58.2)	91 (81.2)	<.001

Values are presented as n (%) or median (IQR). *DANE,* Distal anastomotic new-entry tear; *CKD,* chronic kidney disease; *eGFR,* estimated glomerular filtration rate.

**Table 2:** Intraoperative variables

Variable	Non-DANE group	DANE group	*P* value
	N = 297	N = 112	
Aortic root procedure			
Bentall	6 (2.0)	3 (2.7)	.710
Reimplantation	4 (1.3)	4 (3.6)	.223
Remodeling	1 (0.3)	0	>.99
Partial remodeling	8 (2.7)	8 (7.1)	.048
Use of biological glue at the distal anastomosis	144 (48.5)	54 (48.2)	>.99
GRF glue	59 (19.9)	22 (19.6)	>.99
BioGlue	85 (28.6)	32 (28.6)	>.99
Ascending aortic clamping	210 (70.7)	92 (82.1)	.023
Aortic vascular graft size, mm	24 [24-26]	25 [24-26]	.529
Operative time, min	339 [284-416]	371 [306-437]	.03
CPB time, min	188 [159-224]	191 [168-224]	.33
Crossclamp time, min	111 [64-349]	117 [62-371]	.215
HCA time, min	44 [38-55]	47 [36-55]	.998
Selective cerebral perfusion time, min	85 [40-131]	93 [40-129]	.867
Lowest temperature at bladder, °C	27 [26-28]	27 [26-28]	.60
Intraoperative bleeding, mL	1426 [900-2483]	2090 [1340-3151]	<.001
Cannulation site			
Ascending aorta	27 (9.1)	9 (8.0)	.846
Femoral artery	111 (37.4)	53 (47.3)	.071
Subclavian or axillary artery	38 (12.8)	10 (8.9)	.307
Subclavian or axillary and femoral artery	115 (38.7)	35 (31.2)	.169
Other	4 (1.3)	2 (1.8)	.667
Cardiac cardioplegia			
Retrograde	139 (46.8)	49 (43.8)	.656
Selective	4 (1.3)	1 (0.9)	>.99
Retrograde and selective	18 (6.1)	7 (6.2)	>.99
Antegrade and retrograde	134 (45.1)	55 (49.1)	.505
Other	2 (0.7)	0	>.99
Cerebral perfusion			
Selective cerebral perfusion	297 (100)	110 (98.2)	.075
Selective and retrograde cerebral perfusion	0	1 (0.9)	.274
Extent of aortic replacement			
Hemiarch replacement	126 (42.4)	47 (42.0)	>.99
Partial arch replacement	86 (29.0)	40 (35.7)	.189
Total arch replacement	85 (28.6)	25 (22.3)	.214

Values are presented as n (%) or median (IQR). *DANE,* Distal anastomotic new-entry tear; *GRF,* gelatin-resorcin-formalin; *CPB,* cardiopulmonary bypass; *HCA,* hypothermic circulatory arrest.

### Postoperative Outcomes and Complications

The median time for CT evaluation was 7 days (IQR, 6-11 days) postoperatively in both groups (*P =* .827). Postoperative acute kidney injury occurred significantly more frequently in the DANE group (61.6% vs 50.5%, *P =* .046) (Table 3). Detailed comparisons of patient characteristics, operative variables, and postoperative outcomes stratified by the extent of aortic replacement (HAR, PAR, and TAR) are shown in Table E1, Table E2, Table E3.

**Table 3:** Postoperative variables

Variable	Non-DANE group	DANE group	*P* value
	N = 297	N = 112	
Reexploration for bleeding	18 (6.1)	10 (8.9)	.379
Prolonged ventilation >24 h	174 (58.6)	69 (61.6)	.652
Mediastinitis/deep sternal wound infection	8 (2.7)	5 (4.5)	.356
Acute kidney injury	150 (50.5)	69 (61.6)	.046
Renal replacement therapy	11 (3.7)	8 (7.1)	.185
Tracheotomy	25 (8.4)	8 (7.1)	.839
Postoperative LOS, d	21 [14-31]	25 [15-34]	.192
Postoperative CT for DANE analysis, d	7 [6-11]	7 [5-11]	.827
In-hospital mortality	9 (3.0)	6 (5.4)	.254

Values are presented as n (%) or median (IQR). *DANE,* Distal anastomotic new-entry tear; *LOS,* length of stay; *CT,* computed tomography.

### Location of Distal Anastomotic New-Entry Tear and Long-term Outcomes

**Figure 2:**
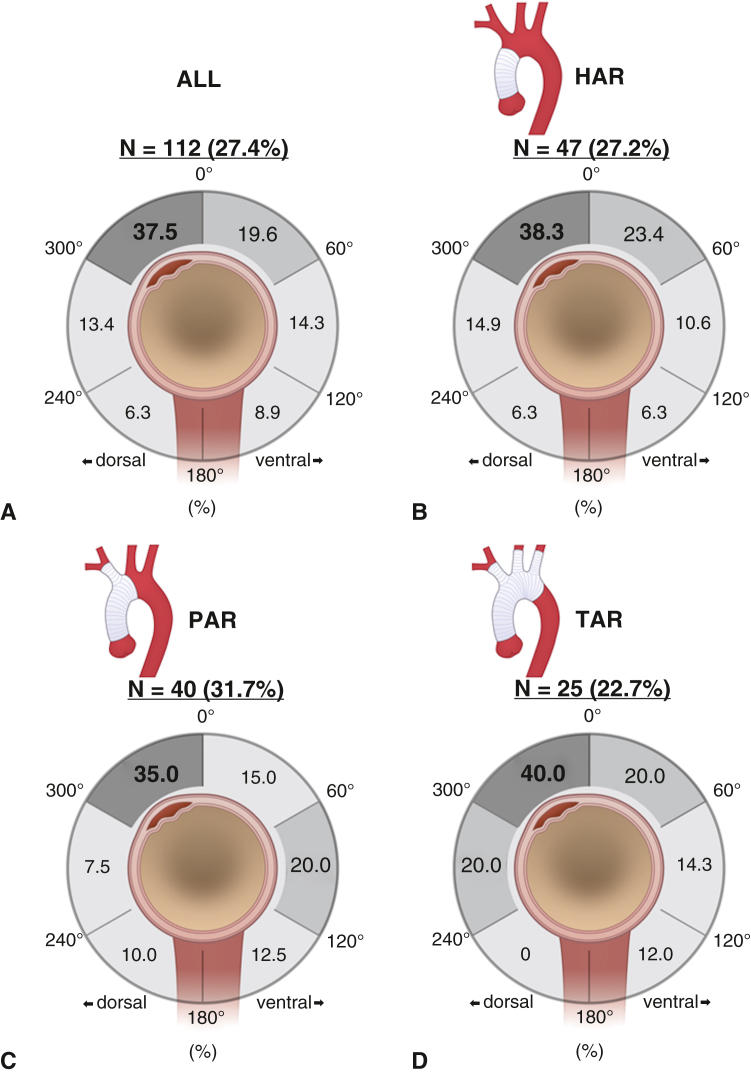
Angular distribution of DANE location shown as the percentage of cases in each 60° sector. A, Overall distribution across all aortic replacement types (n = 112). B, Distribution in the HAR group (n = 47). C, Distribution in the PAR group (n = 40). D, Distribution in the TAR group (n = 25). Note the predominance of DANE in the 301° to 360° position across all replacement types. *HAR,* Hemiarch replacement; *PAR,* partial arch replacement; *TAR,* total arch replacement.

**Figure 3:**
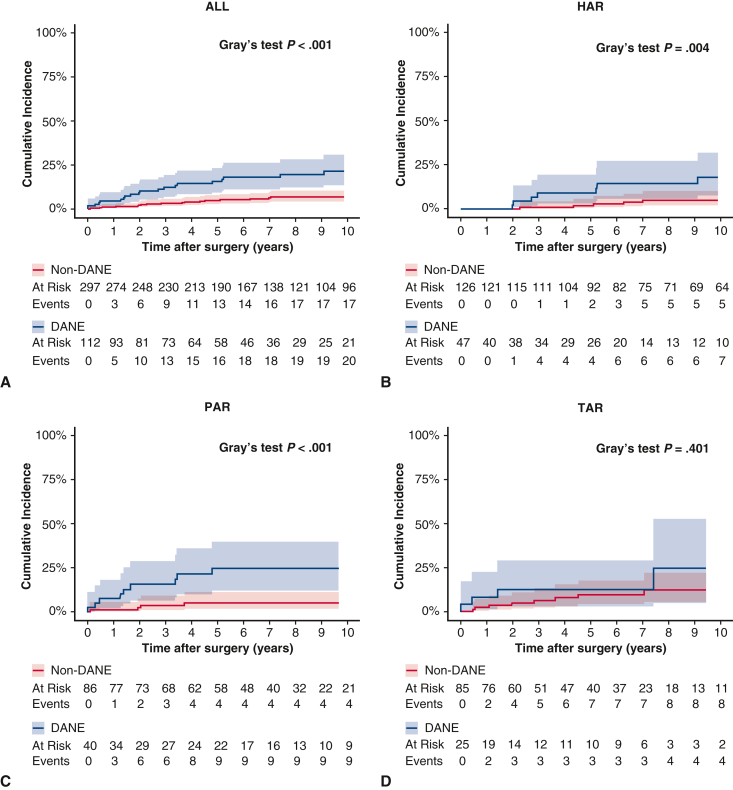
Competing risk analysis of distal aortic reoperation with death as a competing event, stratified by DANE status. The 95% CIs are shown as shaded areas. A, Overall cohort showing significantly higher reoperation rates in patients with DANE (*P < .*001). B, HAR subgroup analysis (*P =* .004). C, PAR subgroup analysis (*P < .*001). D, TAR subgroup analysis showing no significant difference between DANE and non-DANE patients (*P =* .401). *Blue shading* indicates DANE group; *red shading* indicates non-DANE group.. Numbers at risk and cumulative events are shown below each graph. *HAR,* Hemiarch replacement; *PAR,* partial arch replacement; *TAR,* total arch replacement.

**Figure E1:**
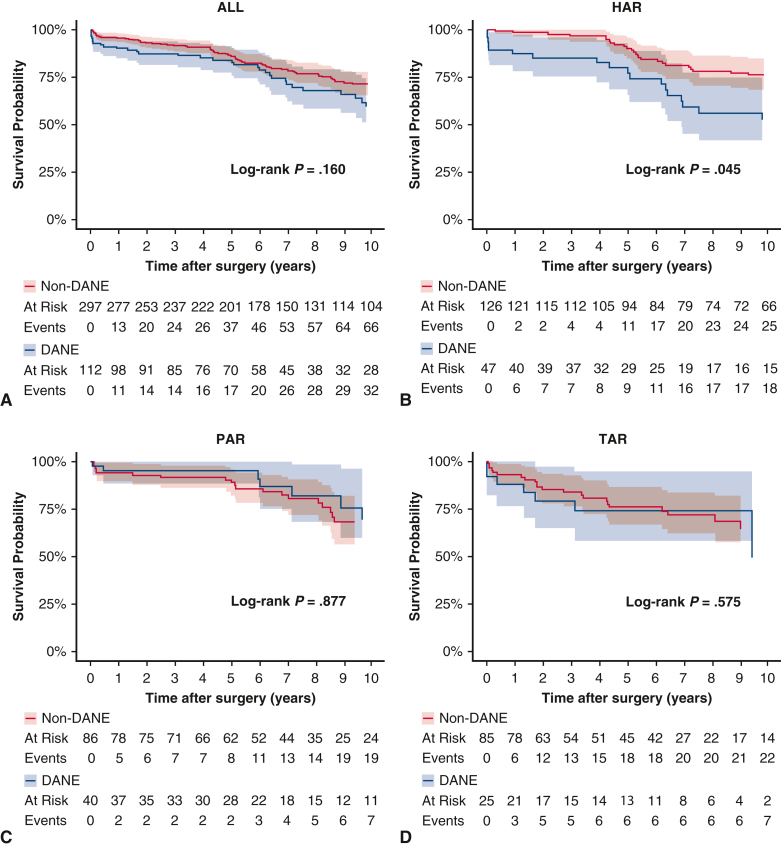
Kaplan–Meier survival curves stratified by DANE status and extent of aortic replacement. The 95% CIs are shown as shaded areas. A, Overall survival comparison between DANE and non-DANE groups showing comparable survival (log-rank *P =* .160). B, Survival in HAR subgroup showing significantly lower survival in patients with DANE (log-rank *P =* .045). C, Survival in PAR subgroup showing no significant difference (log-rank *P =* .877). D, Survival in TAR subgroup showing no significant difference (log-rank *P =* .575). *Blue shading* indicates DANE group; *red shading* indicates non-DANE group.. Numbers at risk and cumulative events are shown below each graph. *HAR,* Hemiarch replacement; *PAR,* partial arch replacement; *TAR,* total arch replacement.

The median follow-up was 6.8 years (IQR, 3.8-10.7 years). Among the 112 patients with DANE (27.4% overall incidence), the predominant location across all extents of aortic replacement was the 301° to 360° position (37.5%, n = 42), followed by the 1° to 60° position (19.6%, n = 22) (Figure 2, *A*). When stratified by specific aortic replacement extent, this pattern remained consistent. In the HAR group (n = 47, 27.2% incidence), DANE occurred most frequently at the 301° to 360° (38.3%, n = 18) and 1° to 60° (23.4%, n = 11) positions (Figure 2, *B*). Likewise, in the PAR group (n = 40, 31.7% incidence), DANE was predominantly observed at the 301° to 360° position (35.0%, n = 14), followed by the 1° to 60° and 121° to 180° positions (20.0%, n = 8 each) (Figure 2, *C*). The TAR group (n = 25, 22.7% incidence) demonstrated the highest prevalence of DANE at the 301 to 360° position (40.0%, n = 10), followed by the 1° to 60° (20.0%, n = 5) position (Figure 2, *D*). Long-term survival analysis revealed comparable overall survival between the DANE and non-DANE groups (log-rank *P =* .16) (Figure E1). The cumulative incidence of distal aortic reoperation was significantly higher in the DANE group than in the non-DANE group (*P* < .001; Figure 3, *A*). The DANE group showed increased reoperation rates, particularly during the first 5 years postoperatively. When stratified by the extent of replacement, DANE was found to significantly increase the distal reoperation rates in HAR (*P =* .004, Figure 3, *B*) and PAR (*P < .*001, Figure 3, *C*) groups. The TAR group showed no significant difference in the reoperation rates between patients with and without DANE (*P =* .401, Figure 3, *D*).

### Risk Factors for Distal Anastomotic New-Entry Tear Occurrence and Distal Aortic Reoperation

The use of biological glue at the distal anastomosis did not influence DANE occurrence (27.3% with glue vs 27.5% without, *P* > .99). In the HAR subgroup, glue use similarly showed no association with DANE (28.3% with glue vs 25.9% without, *P =* .864). Multivariable regression analysis identified supra-aortic branch dissection as the only significant independent risk factor for DANE occurrence (OR, 2.80, 95% CI, 1.60-4.89, *P < .*001). HAR showed a trend toward increased DANE risk, although it did not reach statistical significance (OR, 1.59, 95% CI, 0.86-2.96, *P =* .140).

The multivariable Cox proportional hazards analysis revealed that the presence of DANE (HR, 3.95, 95% CI, 2.05-7.61, *P < .*001) and Marfan syndrome (HR, 7.46, 95% CI, 1.82-30.6, *P =* .005) were independent predictors of distal aortic reoperation (Table 4). Analysis of surgeon volume showed a similar incidence of DANE between high-volume and other surgeons (27.9% vs 26.9%, *P =* .825; Table E4).

**Table 4:** Multivariable analysis of risk factors for distal anastomotic new-entry tear occurrence and distal aortic reoperation

Variable		*P* value
DANE∗	OR (95% CI)	
Age (y)	0.99 (0.97-1.01)	.329
Sex male	1.49 (0.89-2.50)	.133
Body surface area	0.93 (0.55-1.60)	.802
Marfan syndrome	2.65 (0.29-24.5)	.390
Use of glue at distal anastomosis	0.95 (0.60-1.50)	.828
Supra-aortic branch dissection	2.80 (1.60-4.89)	<.001
Hemiarch replacement	1.59 (0.86-2.96)	.140
Partial arch replacement	1.50 (0.80-2.81)	.203
Ascending aortic clamping	1.53 (0.86-2.73)	.148
Distal reoperation†	HR (95% CI)	
Age (y)	0.98 (0.96-1.01)	.215
Sex male	1.69 (0.82-3.48)	.153
Marfan syndrome	7.46 (1.82-30.6)	.005
Use of glue at distal anastomosis	1.56 (0.83-2.93)	.169
Supra-aortic branch dissection	1.00 (0.49-2.04)	.998
Total arch replacement	1.27 (0.56-2.88)	.576
Elephant trunk	2.08 (0.62-6.99)	.235
DANE	3.95 (2.05-7.61)	<.001

*DANE,* Distal anastomotic new-entry tear; *OR,* odds ratio; *HR,* hazard ratio.

∗ OR for DANE.

† HR for distal aortic reoperation.

## Discussion

This comprehensive analysis of 409 patients with ATAAD demonstrated a consistent anatomic pattern of DANE occurrence across different extents of aortic replacement, revealing crucial implications for surgical strategies and long-term surveillance. First, DANE predominantly occurred at the 301° to 360° position across all extents of aortic replacement (37.5% overall, 38.3% in HAR, 35.0% in PAR, and 40.0% in TAR). Second, DANE significantly increased the rate of distal aortic reoperation in the overall cohort (*P < .*001). Third, although DANE increased the reoperation rates in the HAR (*P =* .004) and PAR (*P < .*001) groups, no significant difference in reoperation rates was observed in the TAR group (*P =* .401). Fourth, the multivariable analysis identified supra-aortic branch dissection as the sole independent predictor of DANE occurrence (OR, 2.80, 95% CI, 1.60-4.89, *P < .*001).

The predominant location of DANE at the greater curve of the aortic arch (301°-360°) across all replacement types is a novel finding with significant surgical implications. This consistent pattern suggests that anatomic and biomechanical factors play crucial roles in DANE development. The greater curve of the aortic wall typically experiences greater tension as a result of its concave curvature and pulsatile blood flow patterns against the inner curve of the aortic arch.^8^^,^^9^ This mechanical stress may predispose this region to anastomotic failure, particularly in the setting of fragile dissected tissue. Another reason for the prevalence of DANE in the 301° to 360° position may be the inclusion of DANEs from supra-aortic branches within 2 cm of the distal anastomosis, likely caused by suture injury rather than reentry tears.

When an entry tear is located near the boundary of the replacement area, surgeons determine the extent of replacement. If the surgeon replaces the boundary just proximal to the entry tear, the anastomotic suture line often necessarily incorporates areas adjacent to the supra-aortic branches. This can lead to the suturing of the thinner supra-aortic branch wall, causing cutting through the tissue and subsequent DANE formation. This risk is particularly pronounced when the supra-aortic branches are affected by dissection (Table 1).

Identification of supra-aortic branch dissection as an independent predictor of DANE was previously reported by Ahmad and colleagues,^5^ who found this anatomic factor to be a significant risk factor for DANE. When dissection involves the supra-aortic branches, anastomosis may be performed in more extensively diseased tissues with compromised healing potential. Previous studies have predominantly focused on technical factors, such as anastomotic methods^10^ and biological glue use,^11^^,^^12^ whereas our findings emphasize the importance of preexisting anatomic factors, which may require additional consideration during surgical planning—such as extending the replacement area to ensure more stable tissue for suturing.

The prognostic significance of DANE requires nuanced interpretation. Although DANE remained an independent predictor in multivariable analysis, we acknowledge that it may also reflect the underlying severity of aortic pathology. The higher rates of malperfusion and supra-aortic branch dissection in patients with DANE suggest a more aggressive disease phenotype. However, the anatomic presence of a new communication between the true and false lumens because the distal anastomosis may itself contribute to adverse remodeling by sustaining false lumen pressurization. Thus, DANE likely functions as a marker of disease severity and a mechanical factor influencing outcomes. This dual nature is important for understanding long-term outcomes after ATAAD repair. The clinical application of our findings should be approached with caution. Although supra-aortic branch dissection was identified as a risk factor for DANE, it alone may not be sufficient to guide preoperative planning. The primary determinants of surgical extent—such as entry tear location, extent of dissection, and clinical presentation—remain paramount. Nonetheless, our findings provide valuable insights that may inform surgical decision-making in selected high-risk cases. The results showed that the DANE group had a longer operation time and greater blood loss than the non-DANE group, despite no significant differences in the cardiopulmonary bypass time (Table 2). This suggests that patients with DANE require additional hemostasis time, possibly because of extra suturing. Despite improved hemostatic techniques,^13^ DANE may extend operation time. In addition, residual false lumen flow from DANE may influence postoperative acute kidney injury (Table 3).

The most clinically significant finding of our study was the differential impact of DANE on reoperation rates depending on the extent of aortic replacement. Although DANE significantly increased the distal reoperation rates in the HAR and PAR groups, no significant difference was observed in the TAR group. This observation suggests a potential protective effect of extensive initial surgery in patients with DANE. Several factors may explain this phenomenon. Primarily, the TAR procedure effectively eliminates all dissected aortic arch tissue, which reduces reoperation rates for the residual arch aorta compared with HAR or PAR procedures, where portions of the native arch remain.^14^^,^^15^ Additionally, the ET technique occasionally implemented during TAR procedures may confer enhanced structural stability to the distal anastomosis. In our clinical experience, within the TAR group, the ET technique was used in 33 cases (conventional ET, 11 cases; frozen elephant trunk [FET], 22 cases), and no cases of DANE were observed. The use of FET may prevent DANE and consequently prevent distal reoperations. Interestingly, Yoshitake and colleagues^16^ reported that the distal thoracic reintervention rates did not differ between the FET and no-FET groups, possibly because although FET reduces DANE, it may also lead to distal stent graft–induced new entry (d-SINE), a complication specific to FET. Hiraoka and colleagues^17^ reported that the incidence of d-SINE associated with FET use for aortic dissection was 14.1%, suggesting that the use of FET shifted the problem from DANE to d-SINE by moving the entry point more peripherally. Selecting an optimal FET size may reduce the risk of d-SINE.^18^^,^^19^

Recently, the Ascyrus Medical Dissection Stent (AMDS; Artivion) was introduced for HAR. Based on their propensity score–matched study, AMDS implantation demonstrated a significantly lower incidence of DANE at 17% compared with 45% in the hemiarch group.^20^ This finding aligns with the PERSEVERE trial results, which reported the complete absence of DANE in their AMDS cohort.^21^ The prevention of DANE is clinically significant because these entry tears often lead to persistent false lumen perfusion, negative aortic remodeling, and increased risk of future aortic events.

### Limitations

Our study had certain limitations that warrant consideration. First, despite the large sample size, this was a single-center retrospective analysis with an inherent selection bias. Second, surgical techniques and perioperative management evolved over the 20-year study period,^13^ potentially introducing temporal confounders, and we did not analyze the impact of specific surgical details, such as suture techniques, suture materials, or individual surgeon experience, which may influence DANE occurrence. However, despite temporal variations in surgical approaches, our anastomotic technique remained consistent throughout the 20-year period, with uniform implementation of the turn-up anastomosis methodology in almost all cases. This procedural homogeneity, executed by a cohesive group of 15 surgeons, mitigated potential technique-related confounding variables in our analysis. Last, comprehensive genetic testing was not routinely performed, limiting our ability to evaluate the interaction between genetic predisposition and DANE development. Future studies incorporating systematic genetic assessments may offer further insights into the biological underpinnings of DANE formation.

## Conclusions

DANEs occur predominantly at the greater curve of aortic arch (301°-360°) across all replacement extents and significantly increase the distal reoperation risk in patients undergoing HAR and PAR, but not in patients undergoing TAR. Supra-aortic branch dissection is a key anatomic predictor of DANE, suggesting that preoperative anatomic assessment is crucial for surgical planning. These findings highlight the complex relationship among anatomic features, surgical extent, and long-term outcomes in ATAAD, emphasizing the need for individualized surgical strategies and vigilant postoperative surveillance.

### Webcast

You can watch a Webcast of this AATS meeting presentation by going to: https://www.aats.org/resources/incidence-patterns-and-long-te-9522.

**Figure:**
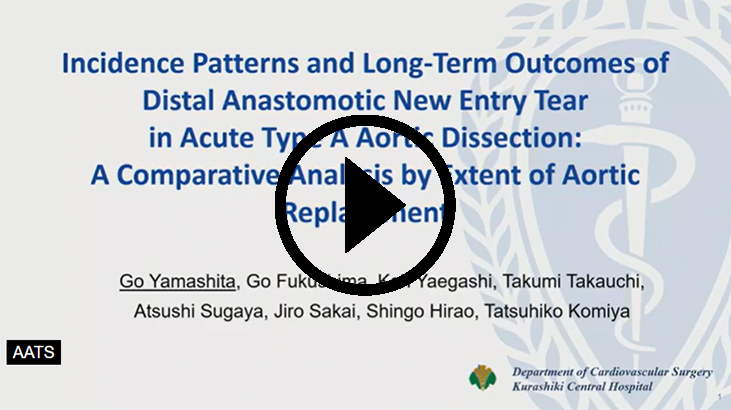

## Conflict of Interest Statement

The authors reported no conflicts of interest.

The *Journal* policy requires editors and reviewers to disclose conflicts of interest and to decline handling or reviewing manuscripts for which they may have a conflict of interest. The editors and reviewers of this article have no conflicts of interest.

## Figures and Tables

**Table E1:** Patient characteristics stratified by extent of aortic replacement

Variable	HAR	PAR	TAR	*P* value
	N = 173	N = 126	N = 110	
Age, y	72 [63-80]	69 [62-77]	62 [52-73]	<.001
Sex (male)	70 (40.5)	59 (46.8)	74 (67.3)	<.001
Body surface area, m^2^	1.57 [1.47-1.73]	1.62 [1.48-1.83]	1.74 [1.56-1.93]	<.001
Body mass index, kg/m^2^	22.9 [20.8-25.4]	22.8 [20.9-25.5]	24.1 [21.4-27.5]	.017
Hypertension	123 (71.1)	82 (65.1)	92 (83.6)	.005
Hyperlipidemia	35 (20.2)	28 (22.2)	15 (13.6)	.216
Smoking	50 (28.9)	46 (36.5)	51 (46.4)	.011
Diabetes	12 (6.9)	5 (4.0)	4 (3.6)	.366
CKD (eGFR <45, mL/min/1.73 m^2^)	56 (32.4)	19 (15.1)	22 (20.0)	.001
Previous stroke	19 (11.0)	11 (8.7)	11 (10.0)	.815
Marfan syndrome	1 (0.6)	2 (1.6)	1 (0.9)	.679
Cardiac tamponade	47 (27.2)	21 (16.7)	11 (10.0)	.001
Cardiogenic shock	35 (20.2)	18 (14.3)	7 (6.4)	.006
Cardiopulmonary resuscitation	6 (3.5)	2 (1.6)	1 (0.9)	.307
Preoperative intubation	8 (4.6)	5 (4.0)	5 (4.5)	.960
Preoperative aortic valve regurgitation				
None or trace	0.308	0.308	0.308	.308
Mild	47 (27.2)	43 (34.1)	25 (22.7)	.142
Moderate	11 (6.4)	8 (6.3)	11 (10.0)	.456
Severe	12 (6.9)	8 (6.3)	5 (4.5)	.709
Malperfusion				
Coronary	8 (4.6)	12 (9.5)	5 (4.5)	.158
Cerebral	12 (6.9)	19 (15.1)	14 (12.7)	.067
Visceral	7 (4.0)	12 (9.5)	14 (12.7)	.025
Lower extremity	14 (8.1)	14 (11.1)	20 (18.2)	.035
Supra-aortic branch dissection	91 (52.6)	102 (81.0)	71 (64.5)	<.001

Values are presented as n (%) or median (IQR). *HAR,* Hemiarch replacement; *PAR,* partial arch replacement; *TAR,* total arch replacement; *CKD,* chronic kidney disease; *eGFR,* estimated glomerular filtration rate.

**Table E2:** Intraoperative variables stratified by extent of aortic replacement

Variable	HAR	PAR	TAR	*P* value
	N = 173	N = 126	N = 110	
Aortic root procedure	20 (11.6)	6 (4.8)	8 (7.3)	.099
Bentall	5 (2.9)	3 (2.4)	1 (0.9)	.534
Reimplantation	5 (2.9)	0	3 (2.7)	.162
Remodeling	1 (0.6)	0	0	.505
Partial remodeling	9 (5.2)	3 (2.4)	4 (3.6)	.455
Use of biological glue at distal anastomosis	92 (53.2)	56 (44.4)	50 (45.5)	.207
GRF glue	58 (33.5)	9 (7.1)	14 (12.7)	<.001
BioGlue	34 (19.7)	47 (37.3)	36 (32.7)	.002
Aortic vascular graft size, mm	26 [24-26]	26 [24-26]	24 [22-26]	.003
Operative time, min	314 [267-384]	341 [298-413]	413 [333-489]	<.001
CPB time, min	173 [150-200]	187 [166-217]	214 [186-260]	<.001
Crossclamp time, min	100 [64-258]	111 [72-335]	144 [62-371]	<.001
HCA time, min	38 [34-46]	46 [41-53]	58 [46-67]	<.001
Selective cerebral perfusion time, min	40 [32-57]	109 [72-134]	140 [101-165]	<.001
Lowest temperature at bladder, °C	27 [26-28]	28 [26-28]	27 [25-28]	.155
Intraoperative bleeding, mL	1506 [849-2405]	1538 [1081-2531]	2028 [1081-3558]	.002
Cannulation site				
Ascending aorta	14 (8.1)	12 (9.5)	10 (9.1)	.904
Femoral artery	73 (42.2)	51 (40.5)	40 (36.4)	.618
Subclavian or axillary artery	21 (12.1)	9 (7.1)	18 (16.4)	.088
Subclavian or axillary and femoral artery	58 (33.5)	54 (42.9)	38 (34.5)	.220
Other	3 (1.7)	0	3 (2.7)	.205
Cardiac cardioplegia				
Retrograde	7 (4.0)	12 (9.5)	14 (12.7)	.025
Selective	14 (8.1)	14 (11.1)	20 (18.2)	.035
Retrograde and selective	10 (5.8)	6 (4.8)	9 (8.2)	.534
Antegrade and retrograde	103 (59.5)	55 (43.7)	31 (28.2)	<.001
Other	1 (0.6)	1 (0.8)	0 (0.0)	.667
Cerebral perfusion				
Selective cerebral Perfusion	172 (99.4)	126 (100.0)	109 (99.1)	.593
Selective and retrograde cerebral perfusion	0	0	1 (0.9)	.256

Values are presented as n (%) or median (IQR). *HAR,* Hemiarch replacement; *PAR,* partial arch replacement; *TAR,* total arch replacement; *GRF,* gelatin-resorcin-formalin; *CPB,* cardiopulmonary bypass; *HCA,* hypothermic circulatory arrest.

**Table E3:** Postoperative variables stratified by extent of aortic replacement

Variable	HAR	PAR	TAR	*P* value
	N = 173	N = 126	N = 110	
Reexploration for bleeding	8 (4.6)	11 (8.7)	9 (8.2)	.305
Prolonged ventilation >24 h	105 (60.7)	68 (54.0)	70 (63.6)	.288
Mediastinitis/deep sternal wound infection	4 (2.3)	5 (4.0)	4 (3.6)	.722
Acute kidney injury	90 (52.0)	69 (54.8)	60 (54.5)	.876
Renal replacement therapy	9 (5.2)	6 (4.8)	4 (3.6)	.875
Tracheotomy	12 (6.9)	11 (8.7)	10 (9.1)	.760
Postoperative LOS, d	20 [14-30]	21 [14-32]	23 [17-34]	.119
Postoperative CT for DANE analysis, d	7 [6-10]	7 [6-11]	7 [6-11]	.996
DANE occurrence	47 (27.2)	40 (31.7)	25 (22.7)	.315
In-hospital mortality	4 (2.3)	5 (4.0)	6 (5.5)	.119

Values are presented as n (%) or median (IQR). *HAR,* Hemiarch replacement; *PAR,* partial arch replacement; *TAR,* total arch replacement; *LOS,* length of stay; *CT,* computed tomography; *DANE,* distal anastomotic new-entry tear.

**Table E4:** Distal anastomotic new-entry tear incidence by surgeon volume

Variable	High-volume surgeons	Other surgeons	*P* value
	N = 197	N = 212	
DANE occurrence	55 (27.9)	57 (26.9)	.825

Values are presented as n (%). High-volume surgeons were defined as those who performed ≥50 cases during the study period. Two surgeons met this criterion (126 and 71 cases, respectively), accounting for 197 (48.2%) of the total 409 cases. The remaining 13 surgeons performed 212 cases. *DANE,* Distal anastomotic new-entry tear.
